# Role of child maltreatment and gender for bipolar symptoms in young adults

**DOI:** 10.1186/s40345-019-0173-9

**Published:** 2020-03-02

**Authors:** Ida S. Haussleiter, Eva Neumann, Sandra Lorek, Bianca Ueberberg, Georg Juckel

**Affiliations:** 1 Research Institute for Mental Health, Ruhr-University Bochum, LWL-University Hospital Bochum, Alexandrinenstr. 1, 44791 Bochum, Germany; 2 Department of Psychiatry, Ruhr-University Bochum, LWL-University Hospital Bochum, Bochum, Germany

**Keywords:** Bipolar disorder, Depression, Child maltreatment, Emotional abuse, Gender

## Abstract

**Background:**

Child maltreatment has been shown to be associated with a wide range of mental disorders, including bipolar disorders. In this 2-year follow-up study, recollections of emotional, physical and sexual abuse were related to bipolar symptoms, namely depressive, hypomanic and manic symptoms.

**Methods:**

The sample consists of 134 students who took part at five measurement times within the 2-year period. Data were collected with self-report scales.

**Results:**

The results show that recollections of abuse, particularly emotional abuse, were associated with more severe depressive symptoms; this finding, however, only applied to women. Hypomanic and manic symptoms were not associated with recollections of abuse. For hypomanic symptoms, however, a significant decrease over the 2 years was observed.

**Conclusions:**

The findings of this study suggest that recollections of abusive experiences in childhood combined with female gender increase the risk for depression, whereas hypomanic and manic states are probably better predicted by other factors, such as current life circumstances.

## Background

Child maltreatment is defined as harmful behavior of significant others towards a child. Apart from forms that take place among peers (e.g., bullying in school), many children suffer from traumatic events caused by adult persons who are supposed to care for the child but fail to fulfill this role. Such misbehavior is differentiated by active and passive forms (abuse and neglect) and by the personal domain affected (emotional, physical and sexual) (Bernstein et al. 2003).

A growing body of evidence shows that child maltreatment is closely associated with mental diseases. Such evidence has been provided, inter alia, for depressive, anxiety, eating, somatoform, substance abuse and personality disorders (for reviews, see: Carr et al. 2013; Kessler et al. 2010; Vachon et al. 2015).

In recent times, the role of child maltreatment for bipolar disorders has also been explored, showing that these adverse experiences are rather common among patients diagnosed with such disorders. In two studies on bipolar disorders, about half of the patients (51% and 54%) reported experiences of abuse and neglect in childhood (Garno et al. 2005; Leboyer et al. 2007). A further analysis in the study by Garno et al. (2005), differentiating between subtypes of child maltreatment, revealed that emotional abuse is the type of trauma most frequently reported by bipolar patients, with a prevalence rate of 37%, followed by physical abuse (24%), emotional neglect (24%), sexual abuse (21%) and physical neglect (12%).

Childhood trauma is associated with the severity and course of bipolar disorders. It has been shown repeatedly that experiences of abuse and neglect in bipolar patients are associated with an earlier onset of the disease, stronger suicidal tendencies (including an elevated risk for suicide attempts) and more substance abuse (Aas et al. 2016; Daruy-Filho et al. 2011; Etain et al. 2008; Maniglio 2013). Further results point to associations of child maltreatment with a higher number of episodes, rapid cycling, more severe symptoms and an increased likelihood of comorbid mental disorders and psychotic symptoms; these results, however, were not found consistently.

Child maltreatment also seems to be associated with the mental health status between episodes, in times of remission. Etain et al. (2008) found positive correlations between a global score of child maltreatment and two scales for the measurement of emotional instability in a sample of euthymic bipolar patients. A more refined analysis revealed that these correlations were mainly based on strong associations of emotional abuse with emotional instability.

As Alloy et al. (2005) point out, most studies on the associations of bipolar disorders with stressful life events (including childhood trauma) do not distinguish between the types of episodes that are the constituting elements of this disorder. According to the DSM-5 (American Psychiatric Association 2013), bipolar disorders comprise depressive, hypomanic and manic episodes, which occur alternately; the occurrence of one manic episode, however, already justifies the diagnosis of a bipolar disorder. It is possible that depressive, hypomanic, and manic states are associated with child maltreatment in different ways. The differences might be quantitative (i.e., the strength of correlations might differ between the three types of episodes) and qualitative (i.e., the three types might be associated with different subtypes of child maltreatment). Because of the lack of studies considering these possible differences, it is unclear how far depressive, hypomanic and manic states are equally related to adverse childhood experiences.

Unipolar depression, however, has found attention in empirical studies on the role of childhood maltreatment for adult psychopathology. There is compelling evidence that depression is correlated with retrospective assessments of child abuse and neglect, indicating that participants who report such experiences show more severe symptoms of depression than participants who report not being affected by such traumas (Goodman and Brand 2009). Studies based on the differentiation of the five subtypes of child maltreatment have further revealed that emotional traumas are of particular relevance (e.g., Carvalho Fernando et al. 2014; Chapman et al. 2004; Martins et al. 2014; Neumann 2017; Spertus et al. 2003). In all of these studies, emotional abuse alone or both emotional abuse and neglect have been shown to be most strongly correlated with depression in adult life. In a recent review on this topic, Nelson et al. (2017) stated that emotional neglect is the type of childhood trauma most frequently reported by patients diagnosed with a depressive disorder. Furthermore, they found all five subtypes of child maltreatment to be associated with an increased risk for developing a depressive disorder; this risk, however, was highest for patients who reported emotional abuse and/or neglect.

In summary, empirical findings on the role of childhood trauma for depression are numerous and clear; hypomania and mania, however, have rarely been explored in this regard. In one of the rare studies in which depression, hypomania and mania were regarded separately, participants with experiences of physical and/or sexual abuse were shown to score higher on a depression scale, whereas only marginal differences emerged on scales for the measurement of hypomania and mania (Schudlich et al. 2015). Emotional abuse and neglect were not considered in this study.

The present study aims to further clarify the associations of child maltreatment with bipolar symptoms. More specifically, recollections of emotional, physical and sexual abuse in childhood are related to depressive, hypomanic, manic and overall bipolar symptoms. Based on prior findings on depression, it is expected that this variable shows strong associations with emotional abuse in particular. Because of the lack of studies on the associations of hypomania and mania with child maltreatment, reasonable hypotheses on these associations are hard to find; these associations are therefore investigated in an exploratory way.

Moreover, time effects are explored. Wittchen et al. (2003) note that little is known about the natural course of bipolar disorders not only in patient samples, but also in community samples which might include individuals with bipolar symptoms who do not receive psychiatric treatment. According to Wittchen et al. longitudinal studies with such individuals could provide insight into time and age effects of bipolar disorders. The present study addresses this research gap. The sample consists of individuals who were not diagnosed with a mental disorder. The study has a longitudinal design; there were five measurement times within a period of 2 years. This procedure allows exploration of whether depressive, hypomanic, manic and overall bipolar symptoms change with the passing of time. Both increases and decreases in the severity of these symptoms are conceivable.

## Methods

### Sample

The present study is part of a large longitudinal research project on prodromal symptoms of bipolar disorders conducted at the LWL University Clinic of the Ruhr-University Bochum since 2014. The participants of this study were recruited at the Ruhr-University and a link to the study homepage was sent to the 42,718 enrolled students via email. A total of 2968 students agreed to participate and fulfilled the inclusion criteria: age 18–40 years, confirmation that they were not diagnosed with a mental disease and digital signing of the informed consent form. There were five measurement times at intervals of 6 months over a period of 2 years. At the first measurement time 2329 participants provided complete data sets but only 134 participants completely filled out the questionnaires at all five measurement times. These 134 data sets were analyzed in the present study.

The sample consists of 48 men (36%) and 86 women (64%). At the first measurement time they had a mean age of 24.64 years (*SD* = 4.08, range = 18–37), 48 (36%) were singles, 74 (55%) had a romantic partner without being married, 11 (8%) were married and 1 (1%) was divorced. As expected in a student sample, the majority of 127 participants (95%) did not (yet) have children.

### Measures

All data were collected with the following self-report scales.

#### Retrospective measurement of child maltreatment

Recollections of child maltreatment were measured with three items developed by Haussleiter et al. (2018). The items refer to experiences of emotional, physical, and sexual abuse and are worded as follows:

- Have you been abused emotionally in your childhood?
- Have you been abused physically in your childhood?
- Did you have unwanted and/or stressful sexual experiences in your childhood?

The items are responded in a yes/no answer format. Thus, they allow differentiation between participants with and without recollections of traumatic experiences in the three areas.

#### Beck Depression Inventory II (BDI)

The BDI is a 21-item self-report measure for the assessment of severity of depression (Beck et al. 1996, German version by Hautzinger et al. 2009). The items describe the criteria of a depressive episode according to the fourth edition of the *Diagnostic and Statistical Manual for Mental Disorders* (DSM-IV). Each item consists of four statements, coded 0–3, that indicate a range of non-agreement to agreement with the respective symptom. Respondents are instructed to mark the answer option that best describes their mental state in the current week. The BDI score is computed by summing up all the answers. Total scores of 20 or more indicate a depressive episode that is at least moderate.

The BDI showed excellent internal consistency in this study (Cronbach’s alpha for the five measurement times: .93, .92, .92, .93, .92).

#### Hypomania Checklist 32 (HCL)

The HCL serves as a measure of hypomanic symptoms and is available in different languages, including English and German (Angst et al. 2005). The items describe experiences and behaviors that are typical of hypomanic episodes. Respondents are instructed to recall a period of their life in which they were in a good mood, and to assess whether the symptoms described in the items applied to them during that period by selecting a yes or no answer for each item. The number of items answered with a yes is the total score. Scores of 14 or more indicate hypomanic conditions.

The internal consistency of the HCL was satisfactory to good in this study (Cronbach’s alpha for the five measurement times: .73, .82, .85, .87, .87).

#### Altman Self-Rating Mania Scale (ASRM)

The presence and severity of manic symptoms were assessed with the ASRM (Altman et al. 1997; German version by Bräunig et al. 1996). The five items of the scale refer to the last week and are answered on a five-point Likert scale from 0 to 4. Total scores of 5 or more indicate clinically relevant manic states.

The internal consistency of the ASRM was satisfactory to good in the present study, taking into account that the scale consists of only five items (Cronbach’s alpha for the five measurement times: .67, .68, .80, 73, .81).

#### Bochumer Screeningbogen Bipolar (BSB)

The BSB is a German self-report scale for the measurement of bipolar symptoms (Zeschel et al. 2013). The 17 items of this measure describe (hypo)manic (e.g., physical agitation, racing thoughts), depressive (e.g., depressed mood, physical exhaustion) and general symptoms (e.g., anxiety, emotional instability). Respondents are instructed to assess the severity of these symptoms on a five-point Likert scale from 0 to 4 with respect to the last week, the last month and a lifetime. In this study, the BSB total score referring to the last month is considered in the analysis. A cut-off-score of 22 is proposed for the distinction between clinically not relevant and relevant bipolar conditions (Haussleiter et al. 2018).

The BSB showed good internal consistency in the present study (Cronbach’s alpha for the five measurement times: .80, .82, .83, .85, .86).

## Results

In the first step of the analysis, tests on normal distribution were conducted. Kolmogorov–Smirnov tests show that the total scores of the BDI, HCL, ASRM and BSB significantly deviate (*p* < .001) from the normal distribution at all five measurement times. For this reason, the data were analyzed using non-parametric tests.

To find out whether the participants of this study were a representative sample from the initial pool of participants, differences between dropouts (*n* = 2195) and non-dropouts (*n* = 134) with regard to child abuse and depressive, hypomanic, manic and overall bipolar symptoms assessed at the first measurement time were tested. Chi square tests showed that the non-dropouts did not differ significantly in the frequencies of emotional, physical and sexual abuse from the participants who dropped out of the study (emotional abuse: *χ*^2^(1) = 2.29, n.s.; physical abuse: *χ*^2^(1) = 0.23, n.s.; sexual abuse: *χ*^2^(1) = 1.52, n.s.). Differences between the BDI, HCL, ASRM and BSB scores at time 1 were tested with Mann–Whitney U tests. There were no significant differences with regard to the BDI (*z* = − 1.52, n.s.), HCL (*z* = − 1.31, n.s.) and ASRM (*z* = − 1.04), but the non-dropouts scored slightly lower on the BSB (dropouts: median = 14.00; non-dropouts: median = 12.50; *z* = − 2.31, *p* < .05). In summary, the differences between dropouts and non-dropouts can be considered marginal.

Table 1 shows the distribution of childhood trauma among the 134 participants of this study. There are gender differences in the frequencies of emotional and sexual abuse, with women being affected more often than men. Chi square tests reveal that the difference is significant for sexual abuse, whereas only a non-significant trend (*p* < .10) is shown for emotional abuse.

**Table 1:** Distribution of childhood trauma

Type of trauma	Total		Male		Female		*χ*^*2*^*(1)*
	No	Yes	No	Yes	No	Yes	
	*n (%)*	*n (%)*	*n (%)*	*n (%)*	*n (%)*	*n (%)*	
Emotional abuse	119 (89)	15 (11)	46 (96)	2 (4)	73 (85)	13 (15)	3.72
Physical abuse	122 (91)	12 (9)	44 (92)	4 (8)	78 (91)	8 (9)	0.04
Sexual abuse	121 (90)	13 (10)	47 (98)	1 (2)	74 (86)	12 (16)	4.95*

*p < .05

Next, the course of depressive, hypomanic, manic and overall bipolar symptoms was analyzed. As can be seen from Table 2, the scores for the HCL and BSB decrease from the first to the last measurement time. Friedman tests show that these decreases are significant. For the BDI and ASRM scores no significant changes are observed from the first to the last measurement time.

**Table 2:** Course of depressive, hypomanic, manic and overall bipolar symptoms from Time 1 to Time 5

	Time 1	Time 2	Time 3	Time 4	Time 5	*χ*^*2*^ (4)
	*Mdn*	*Mdn*	*Mdn*	*Mdn*	*Mdn*	
BDI	8	8	8	6	6	8.75
HCL	16	15	14	13	12	73.93***
ASRM	2	2	2	2	1	9.43
BSB	12.5	11	11	10	8	50.23***

*BDI* Beck Depression Inventory, *HCL* Hypomania Checklist, *ASRM* Altman Self-Rating Mania Scale, *BSB* Bochumer Screeningbogen Bipolar, *Mdn* median, ***p < .001

To explore whether bipolar symptoms vary with gender and childhood trauma, differences between men and women and between participants with and without experiences of emotional, physical and sexual abuse in the BDI, HCL, ASRM and BSB scores were tested with Mann–Whitney U tests. Table 3 shows the results.

**Table 3:** Differences in depressive, hypomanic, manic and overall bipolar symptoms depending on gender and childhood trauma

	Gender			Emotional abuse			Physical abuse			Sexual abuse		
	Male	Female	*z*	No	Yes	*z*	No	Yes	*z*	No	Yes	*z*
	*Mdn*	*Mdn*		*Mdn*	*Mdn*		*Mdn*	*Mdn*		*Mdn*	*Mdn*	
Depressive symptoms												
BDI 1	5	9	− 2.36*	7	23	− 4.81***	8	14	− 2.17*	8	17	− 2.49*
BDI 2	5	8	− 2.51*	7	17	− 3.53***	7	16	− 3.03**	7	17	− 2.54*
BDI 3	4	9	− 2.91**	7	17	− 4.06***	7	17	− 2.24*	7	21	− 3.03**
BDI 4	5	8	− 2.44*	5	14	− 3.07**	6	17	− 1.99*	6	14	− 2.17*
BDI 5	6	6	− 0.99	5	13	− 3.87***	6	15	− 2.82**	6	19	− 3.35**
Hypomanic symptoms												
HCL 1	16	16	− 0.36	16	17	− 1.23	16	17	− 1.33	16	18	− 2.08*
HCL 2	15	15	− 0.33	15	17	− 0.62	15	18	− 1.18	15	17	− 1.96
HCL 3	14	14	− 0.12	14	15	− 1.98*	14	16	− 0.49	14	15	− 1.40
HCL 4	13	13	− 0.05	13	16	− 1.03	13	18	− 1.48	13	17	− 2.24*
HCL 5	12	12	− 0.03	12	16	− 1.05	12	17	− 1.33	12	17	− 2.01*
Manic symptoms												
ASRM 1	2	2	− 0.96	2	2	− 1.19	2	2	− 0.46	2	2	− 0.16
ASRM 2	2	2	− 0.14	2	3	− 0.97	2	3	− 0.38	2	2	− 0.29
ASRM 3	2	2	− 1.19	2	5	− 1.97*	2	3	− 0.69	2	2	− 1.75
ASRM 4	2	1	− 0.14	2	3	− 1.39	2	3	− 1.37	2	3	− 1.41
ASRM 5	2	1	− 1.89	1	1	− 0.21	1	0	− 0.84	1	0	− 2.06*
Overall bipolar symptoms												
BSB 1	10	14	− 2.22*	12	21	− 3.85***	12	17.5	− 1.14	12	15	− 1.85
BSB 2	10	11	− 1.25	10	17	− 2.99**	10.5	12.5	− 0.25	11	11	− 0.65
BSB 3	9	12	− 1.93	11	22	− 3.54***	11	12.5	− 0.54	11	18	− 2.29*
BSB 4	8	12	− 2.55*	9	20	− 3.36**	10	11	− 1.36	10	14	− 2.12*
BSB 5	8	8	− 0.81	8	17	− 3.55***	8	7.5	− 1.02	8	12	− 1.85

*BDI* Beck Depression Inventory, *HCL* Hypomania Checklist, *ASRM* Altman Self-Rating Mania Scale, *BSB* Bochumer Screeningbogen Bipolar*, Mdn* median, *p < .05, **p < .01, ***p < .001

Depressive symptoms, in particular, show clear differences between the groups. Men and women differ significantly in the BDI scores at four of the five measurement times, with women scoring higher on that scale. Significant differences also occur with regard to all three types of child abuse. Participants who report experiences of emotional, physical and sexual abuse score higher on the BDI at all five measurement times than participants without such experiences.

In contrast, hypomanic and manic symptoms do not vary considerably with gender and childhood trauma. With few exceptions, the Mann–Whitney U tests do not show significant group differences for the HCL and ASRM scores.

With regard to overall bipolar symptoms, gender differences also appear as minor, as there are no significant differences between men and women at three of the five measurement times. While participants with and without experiences of physical and sexual abuse do not differ significantly in the BSB scores in most cases, there are clear differences between emotionally abused and not abused participants, with emotionally abused participants scoring higher on the BSB at all five measurement times.

**Fig. 1:**
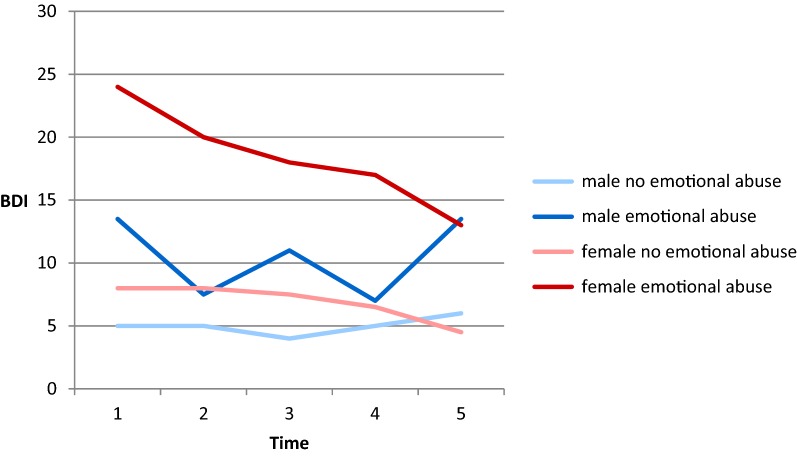
Course of depressive symptoms from Time 1 to Time 5 for men and women with and without experiences of emotional abuse. *BDI* Beck Depression Inventory

**Fig. 2:**
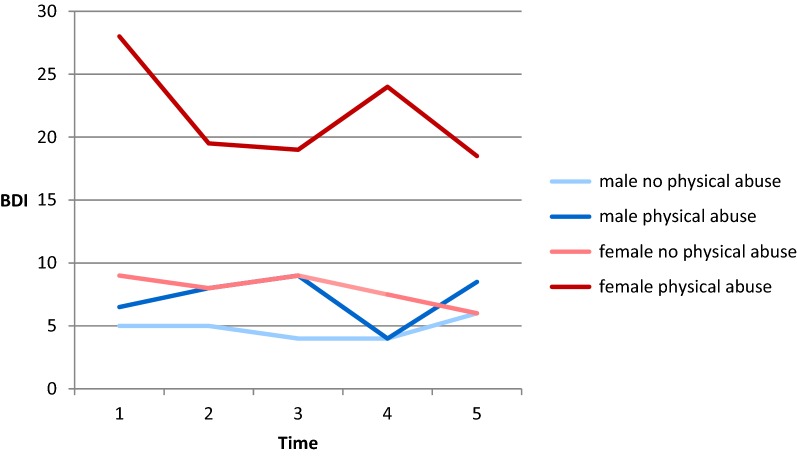
Course of depressive symptoms from Time 1 to Time 5 for men and women with and without experiences of physical abuse. *BDI* Beck Depression Inventory

The strong relations of depressive symptoms with gender and childhood trauma that were found in the foregoing analysis were further explored. With respect to emotional and physical abuse, four groups were compared with each other, having gender as one grouping variable and the statement of being affected or not affected by the respective trauma as the other. (This analysis was not conducted for sexual abuse because the group of men with recollections of sexual abuse consisted of only one person.) The median values of depressive symptoms in the four groups are shown in Figs. 1 and 2. With only one exception, women with experiences of emotional and/or physical abuse have the highest BDI scores of all groups. Kruskal–Wallis tests show that the differences between the four groups are significant in all cases (emotional abuse: BDI 1: *χ*^*2*^ (3) = 25.87, *p* < .001; BDI 2: *χ*^*2*^ (3) = 17.02, *p* < .01; BDI 3: *χ*^*2*^ (3) = 21.89, *p* < .001; BDI 4: *χ*^*2*^ (3) = 14.04, *p* < .01; BDI 5: *χ*^*2*^ (3) = 15.27, *p* < .01; physical abuse: BDI 1: *χ*^*2*^ (3) = 12.08, *p* < .01; BDI 2: *χ*^*2*^ (3) = 15.11, *p* < .01; BDI 3: *χ*^*2*^ (3) = 13.68, *p* < .01; BDI 4: *χ*^*2*^ (3) = 9.75, *p* < .05; BDI 5: *χ*^*2*^ (3) = 8.80, *p* < .05).

## Discussion

This study shows that subclinical conditions of bipolar symptoms are associated with recollections of child maltreatment in specific ways. While hypomanic and manic symptoms do not differ depending on recollections of abuse, depressive symptoms are clearly higher among participants who report abusive experiences. This difference was found for all three types of child maltreatment considered in this study (i.e., emotional, physical and sexual abuse) but the strongest effect appeared with regard to emotional abuse. Reports of emotional abuse in childhood were associated with high depression scores. This result corresponds to former studies on the relations of child maltreatment with depression in adult life.

Further analysis revealed that experiences of abuse cannot be regarded solely as risk factors for depressive symptoms in adult life. When gender was included in the analyses, it became apparent that only women who report experiences of abuse score higher on the depression scale. Men scored as low on the depression scale as women without recollections of abuse. Thus, in this study experiences of abuse combined with female gender appear as a risk pattern for depression in adult life, whereas male gender seems to be a protective factor, independent of the presence or absence of recollections of abuse. The moderating effect of gender with respect to the detrimental effects of abuse is a new finding that needs support in future studies.

These associations, however, were only shown for emotional and physical abuse and could not be analyzed with regard to sexual abuse due to the small number of men in the sample who reported being affected by this trauma. The associations of depressive symptoms, sexual abuse and gender therefore need further clarification.

Hypomanic and manic symptoms appeared unaffected by child maltreatment, but for hypomanic symptoms an interesting time effect emerged because they showed a significant decrease over the 2 years of measurement. It is probable that this result reflects an age effect. At the beginning of this study, the participants were young adults in their early 20 s. During the following two years they probably became more mature and it is likely, for example, that their emotional stability increased. This maturing process might have diminished the tendency to go through phases of intense and changing feelings and high activity that are typical of hypomanic states. The effect of time that was found in this study suggests that subclinical conditions of hypomania become less frequent and less pronounced with increasing age. This assumption is supported by findings from a large transcultural study in which significant decreases with age in the HCL scores were consistently found in 12 different countries (Angst et al. 2010).

Manic symptoms were not related to recollections of child maltreatment and nor did they show a time or age effect. Thus, in this study no psychosocial factors could be identified that are associated with this mental state. A possible explanation for this finding is that manic symptoms are not related to past experiences in childhood but to current interpersonal experiences. It might be informative to explore associations of manic symptoms with close relationships in adult life, for example by relating them to the attachment dimension “anxiety”, which stands for intense worries and a strong desire for attention and care in romantic relationships (Neumann et al. 2007). Attachment anxiety has been shown to be associated with many mental disorders (Mikulincer and Shaver 2016) and might also be correlated with mania, particularly as these two constructs are both characterized by strong inadequate feelings and overreacting behavior.

### Limitations

This study has several limitations. First, child maltreatment was assessed with three single items for the measurement of emotional, physical and sexual abuse. This short measure was used for economic reasons; the set of questionnaires should not be too long in this online survey. Even though the measure is quite simple, there is some evidence that the three items led to reliable results. Iffland et al. (2013) used the Childhood Trauma Questionnaire (CTQ) by Bernstein et al. (2003) in a large representative sample from the German population (*N* = 2500). The prevalence rates found in this study (emotional abuse: 10%; physical abuse: 12%; sexual abuse: 6%) are close to the rates of the present study (emotional abuse: 11%; physical abuse: 9%; sexual abuse: 10%). Furthermore, Iffland et al. also found a higher frequency of sexual abuse among women. Although these similarities suggest that child abuse was measured adequately in this study, the use of a multi-item scale such as the CTQ should be preferred in future studies.

Second, the measurement of child maltreatment was retrospective. The validity of retrospective measures is a controversial issue and has been discussed intensely. Hardt and Rutter (2004) recommend handling retrospective data with skepticism because of several limitations of this approach. For example, there are findings showing that documented occurrences of sexual abuse in childhood are not reported in adulthood by many persons concerned, particularly men. This phenomenon might have played a role in this study; it is striking, in any case, that only one man reported sexual abuse in childhood. Because of the retrospective nature of the data on child maltreatment, the findings of this study need support from data obtained in childhood.

Third, there was a substantial loss of participants from the first to the last measurement. Only 6% of the initial sample provided complete data sets for each measurement time and it is assumed that these participants differ from those who dropped out of the study; they are probably more reliable and better organized. This personality structure might rely on good mental health and little stress in childhood or in present life. Thus, it cannot be ruled out that the sample of this study is not representative for student populations; it is possible that it mainly consists of healthy persons from low-risk environments.

Fourth, in line with the foregoing arguments, the participants were not diagnosed with a bipolar disorder and the median scores for depressive, hypomanic, manic and overall bipolar symptoms were below the cut-off scores for clinically relevant levels. These results were to be expected, because the participants of this study were students, not patients, who had to confirm that they were not diagnosed with a mental disorder. It would be interesting to explore whether the findings of this study can be replicated in clinical samples of patients with a bipolar disorder.

## Conclusions

In this study the constituent elements of bipolar disorders, depressive, hypomanic and manic symptoms, were explored separately with regard to their relations with recollections of child abuse. This differentiated analysis revealed that only depressive symptoms are associated with child abuse, especially with emotional abuse. However, this result was only found for women, which might indicate that child abuse is a risk factor for depression in adulthood for women but not for men. Hypomanic symptoms were not related to recollections of abuse but showed an effect of time, suggesting that hypomanic states become less strong and less frequent with increasing age. Manic symptoms did not show any associations with the psychosocial factors considered in this study; this mental state is probably determined more by other factors such as current life stressors.

## Data Availability

The data sets generated and analyzed during the current study are available from the corresponding author by reasonable request.
